# Characterization of the dominant strain (G-VII) of Newcastle disease viruses isolated from commercial chickens in Bangladesh during recent outbreaks

**DOI:** 10.5455/javar.2024.k790

**Published:** 2024-06-09

**Authors:** Mohammad Aynul Haque, Mohammad Sadekuzzaman, Md. Enamul Haque, Mst. Kohinoor Parvin, Md. Mostofa Kamal, Sajedul Hayat, Md. Ariful Islam, Mst. Minara Khatun, Mahbubul Pratik Siddique, Sham Soun Nahar, A. K. M. Khasruzzaman, Muhammud Tofazzal Hossain, Md. Alimul Islam

**Affiliations:** 1 Department of Microbiology and Hygiene, Bangladesh Agricultural University, Mymensingh, Bangladesh; 2 Central Disease Investigation Laboratory, Department of Livestock Services, Dhaka, Bangladesh; 3 Department of Microbiology, Sheikh Hasina University of Science and Technology, Bhairab, Bangladesh; 4 Livestock Research Institute, Mohakhali, Dhaka, Bangladesh

**Keywords:** NDV, RT-PCR, ICPI, IVPI, MDT genotype VII

## Abstract

**Objective:**

Newcastle disease virus genotype VII (NDV-GVII), an extremely infectious pathogen, has been causing severe economic consequences for the chicken industry. The current study aimed to isolate and characterize NDV-GVII from commercial chickens in Bangladesh during a recent outbreak.

**Materials and Methods:**

From clinically suspected chickens from 70 commercial poultry farms, a total of 420 samples (trachea, lungs, and brain tissue) were collected. The samples were cultivated in 9–10 day-old seronegative embryonated chicken eggs (ECEs) after evaluating them using the rapid Newcastle disease virus (NDV) antigen detection kit. The hemagglutination (HA) inhibition test, agar gel immune diffusion (AGID) test, molecular detection by reverse transcription-polymerase chain reaction (RT-PCR), and phylogenetic studies using gene sequences of fusion (F) protein. The HA pattern of isolated NDV was determined using different avian and mammalian red blood cells (RBCs). The pathogenicity of the isolated virus was evaluated using mean death time (MDT), intravenous pathogenicity index (IVPI), and intracerebral pathogenicity index (ICPI).

**Results:**

The study found 87 NDV samples positive using the rapid NDV Ag detection kit and then 60 positives for virus isolation in ECEs. All 60 isolates were positive for NDV by HI, AGID, and RT-PCR. Phylogenetic tree analysis indicated that recent NDV isolates belong to genotype VII and exhibit a similarity of 99.7%–98.5% with isolates from Bangladesh, Iran, and India. The new isolates, identified as velogenic strains of NDV, possess an F protein cleavage site with ^112^-R-T-K-R-F-^117^ amino acid motifs. The isolated NDV showed diversified HA activity while using RBCs from birds and mammals. The results of ICPI, IVPI, and MDT indicated that the recent NDV isolates were very virulent.

**Conclusion:**

This study concluded that NDV-GVII is prevalent in commercial poultry farms in Bangladesh.

## Introduction

In livestock farming, the poultry industry is a highly integrated and constantly growing industry and a significant contributor to the agricultural sector of Bangladesh [1]. The poultry population is just around 385.7 million in Bangladesh [2]. By 2021, the country is projected to need 17 billion eggs, 2 million tons of chicken meat, 86 million day-old chicks, and 8 million megatons of feed to fulfill the demand. The estimated annual per capita poultry consumption in the country is around 7 kg for 2020 [3]. The consumption of animal-based proteins, including poultry meat and eggs, is expected to grow substantially for at least the next ten years. To meet the growing domestic demand, substantial investments in enhanced (more knowledge-intensive) production techniques are foreseen. This gives companies and knowledge institutions in almost every part of the poultry value chain good chances to make money. The re-emerging and transboundary infectious viral diseases of poultry continue to be a significant concern for the poultry industry in Bangladesh [4]. Hence, the economic consequences of these diseases for chicken producers and the economy of Bangladesh are considerable [5].

Newcastle disease (ND) is a very consequential viral disease that is resurfacing and can spread globally, impacting poultry populations [6]. The Newcastle disease virus (NDV) is a virus that causes ND, which affects poultry in Bangladesh [7]. NDV has a high susceptibility in chicken populations, and pathogenicity varies by strain. Based on pathogenicity, the strains are divided into three groups: velogenic strains (neurotropic, viscerotropic, and pantropic), mesogenic strains (moderately pathogenic), and lentogenic strains (not harmful at all) [8]. The primary constraint of backyard chicken farming is the potential for outbreaks, which can result in a 100% mortality rate [9] and reduced body mass and egg production in the surviving chickens [10]. The strains of NDV are divided into classes I and II according to the nucleotide sequences of their F and L genes and their genome size [11]. Class II strains of NDVs showed more genetic variety, with 20 new genotypes nominated as I through XXI, excluding XV, which contains recombinant viruses [8]. These genotypes comprise both highly transmissible and non-transmissible strains [12]. A new genotype, NDV genotype VII, has gradually emerged as the primary prevalent strain worldwide [13].

Genotype VII viruses emerged in Southeast Asia during the 1980s and were subsequently disseminated globally, with the exception of Australia and North America [7]. The genotype VII viruses may be further categorized into sub-genotype VII.1.1, sub-genotype VII.1.2, and sub-genotype VII.2 [8].

The genotype VII.1.1 viruses were responsible for the fourth major epidemic of ND. These viruses emerged in the Far East around 1985 and quickly disseminated throughout Asia, Europe, Africa, and the Middle East. Between 2005 and 2010, the viruses of genotype VII.2 were prevalent in Indonesia and Malaysia [8,14]. They subsequently moved to Africa, Europe, the Middle East, and Central and East Asia, resulting in the fifth ND pandemic [14,8]. Field outbreaks of ND are prevalent in Bangladesh. The scientists analyzed velogenic NDV and discovered genotypes XIII.2 in hens [15] and XXI.1.2 in pigeons [16]. According to Nooruzzaman et al. [7], this was the first time that the pathotypically virulent Genotype VII.2 of the NDV was found, characterized, and identified in Bangladesh during 2020–21. This particular strain was shown to be responsible for outbreaks in broiler chickens. Therefore, commercial poultry farms in Bangladesh regard NDV as a significant constraint that not only results in substantial mortality and production losses, but also induces economic losses through trade restrictions.

The average economic loss per family per year owing to the ND outbreak was estimated to be Bangladeshi taka 2,561 [5]. On average, the nation suffered an annual financial loss of US $288.49 million [5]. For this reason, the isolation and identification of virulent NDV genotype VII were required to diagnose the disease. This research is designed to isolate and characterize the most recent samples of NDV from commercial poultry suspected of having ND from several farms in Bangladesh.

## Materials and Methods

### Ethical statement

The experimental study followed the instructions of the Animal Welfare and Experimentation Ethics Committee (AWEEC) of Bangladesh Agricultural University (BAU) in Mymensingh-2202. It received formal approval with the identifier [Ref. No. AWEEC/BAU/2021(51)].

### Study areas

**Figure 1.**
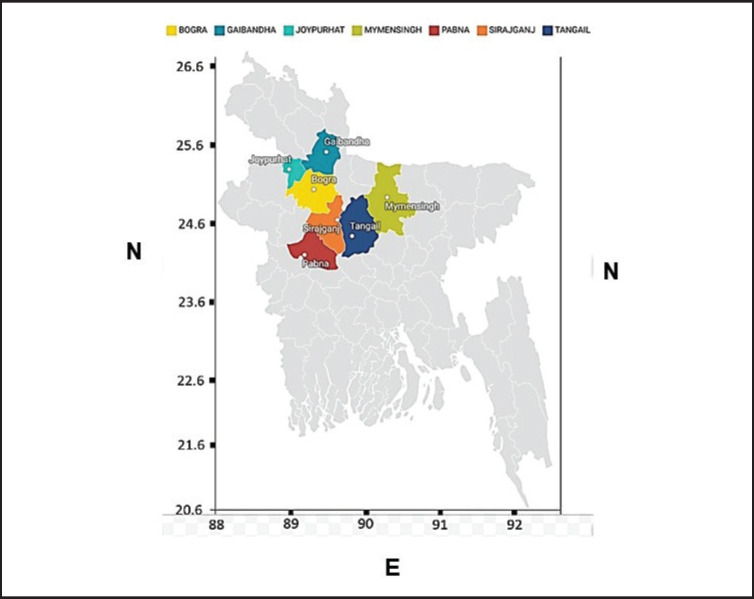
Colored areas in the map show the sampling areas.

The suspected NDV field samples were collected from seven districts in Bangladesh, particularly Joypurhat (25.0968°N, 89.0227°E), Gaibandha (25.3290°N, 89.5415°E), Bogura (24.8526°N, 89.3730°E), Sirajganj (24.3141°N, 89.5700°E), Pabna (24.1585°N, 89.4481°E), Tangail (24.3917°N, 89.9948°E), and Mymensingh (24.7539°N, 90.4073°E) (Fig. 1).

### Collection of samples

In this study, 420 samples were collected from 70 commercial poultry farms suspected of ND outbreaks. These farms are categorized into 23 layer, 18 Sonali, and 29 broiler farms. The collected samples consisted of 138 from layer birds, 108 from Sonali, and 174 from broilers. Samples, including trachea, lungs, and brain tissue, were taken aseptically from birds showing symptoms of ND such as difficulty in breathing, loud coughing, gasping, discharges from the eyes and nostrils, swollen eyelids, loss of appetite, ruffled feathers, lethargy, weakness, tremors, and diarrhea. Subsequently, the samples were sent to the virology laboratory located at BAU in Mymensingh-2202 under the Department of Microbiology and Hygiene. The samples were kept at −80 °C in the refrigerator for further investigation.

### Processing of samples

Field samples were thawed and macerated using a sterilized pestle and mortar. After that, 1X sterile PBS was used to make a 20% (w/v) suspension of the samples. The mixture was centrifuged at 5,000 rpm for 30 min at 4°C to produce a clear solution. Subsequently, the supernatant was collected and treated with antibiotics (2 mg/ml streptomycin, 50 µg/ml gentamycin, 2000 units/ml penicillin, and 1000 units/ml myostatin) to prevent bacterial and fungal infections. To verify the sterility of the inoculum, it was cultivated in a bacterial and fungal medium and incubated at 37°C for 24 h.

### Primary screening by rapid NDV antigen kit

The NDV antigen rapid test kit (Shenzhen Lvshiyuan Biotechnology Company Ltd., Shenzhen, China) was used for the first screening of the samples in accordance with the instructions supplied by the kit’s manufacturer. The outcomes were discernible within 3–5 min, relying on visual inspection for the detection of bands: a single band indicated a negative result, whereas a double band signified the presence of NDV antigen. A positive NDV result was confirmed by the appearance of two-colored bands in the test area designated for NDV antigen detection.

### Isolation of NDV using embryonated chicken eggs (ECEs)

NDV was isolated using 9–10-day-old seronegative chicken eggs in accordance with the protocol outlined in the Office International Epizootics (OIE) guideline [17]. A 0.2-ml sterile inoculum was introduced into each egg via the allantoic cavity route and kept at a temperature of 37°C until mortality was found. Twice daily, the eggs were incubated with candles, and dead embryos were frozen overnight at 4°C. The allantoic fluid was collected from dead embryos. Utilizing 2% cRBC, the slide hemagglutination (HA) test identified the existence of the hemagglutinating virus. The allantoic fluid was sorted at −80°C for further use in serological, molecular, and biological characterization. Hemagglutination inhibition (HI) test tests were performed to detect the recent isolates of NDV using NDV-specific antiserum, according to the OIE manual [17]. Agar gel immunodiffusion (AGID) assay was performed according to Islam et al. [18] to detect the recent isolates of NDV using NDV-specific hyperimmune serum.

### Extraction of viral RNA

Viral RNA was extracted from the allantoic fluid using the RNeasy Mini Extraction Kit (Qiagen, Germany) according to the manufacturer’s instructions. The RNA that was obtained was stored at a temperature of −80°C in anticipation of the polymerase chain reaction (PCR) investigation.

### Reverse transcription-polymerase chain reaction (RT-PCR) reaction

The RT-PCR test was performed in a thermos cycler (BIO-RAD, Singapore) using a reaction mixture volume of 25 µl composed of 2 µl of each forward and reverse primer, 12.5 µl of the master mix (5X RT-PCR buffer), 1 µl of the 25X RT-PCR enzyme mix (AgPath-10^TM^ one-step RT-PCR, Thermo Fisher Scientific, USA), 4.5 µl of nuclease-free water, and 3 µl of template RNA. The thermal cycling conditions were established as follows: a total of 40 PCR cycles were performed, consisting of a 30-sec denaturation at 95°C, a 30-sec annealing at 50°C, and a 42-sec extension at 72°C. These cycles were carried out after a reverse transcription step at 45°C for 60 min. The experiment was concluded by performing a final extension at 72°C for 10 min. Primer pairs NDV_F: 5′-TTG ATG GCA GGC CTC TTG C-3′ and NDV_R: 5′-AGC GT(C/T) TCT GTC TCC T-3′ were utilized to target the F gene of NDV. The PCR amplification products were then evaluated for their expected size of 255 base pair (bp) through 1.5% agarose gel electrophoresis (Mupid^®;^-One system, Japan) and imaged with a gel documentation system (Bio-Rad, USA), as mentioned by Li et al. [19].

### Sequence analysis

The cDNA from three selected ND virus strains was purified and analyzed. The analysis focused on the “F” gene for NDV. These genes were sequenced using a Genetic Analyzer 3,500 (Applied Biosystems, USA). The “F” gene nucleotide sequences from the three ND virus strains were compared with 21 other sequences from GenBank to figure out their phylogenetic relationships. This analysis employed the Neighbor-Joining method, as initially described by Saitou and Nei [20]. The reliability of the resultant phylogenetic tree was assessed using a bootstrap approach involving 1,000 repeats. The frequency of taxa occurrence in these duplicate trees is given alongside the branches, following a methodology introduced by Felsenstein [21]. The evolutionary investigations were carried out by the MEGA 11 program, as detailed by Tamura et al. [22].

### Virulence determination of NDV isolates in avian and mammalian blood by HA test

To determine the HA activity pattern, 50 µl of 2% blood suspension from four avian and eight mammalian species was mixed with an equal volume of allantoic fluid from each isolate. The solution was allowed to stand at ambient temperature for a duration of 5 to 10 min until the appearance of transparent HA on the slide. The interpretation of NDV isolate HA activity was considered strong, moderate, or weak depending on the number of avian and mammalian species displaying HA activity in the blood, following the guidelines of the OIE manual [17].

### Determination of pathogenicity of isolated NDV

The pathogenicity indices of field isolates of NDV were evaluated using a variety of procedures. The experiments performed included the intracerebral pathogenicity index (ICPI) test on one-day-old specific pathogen-free (SPF) chicks and the intravenous pathogenicity index (IVPI) test on 45-day-old SPF chickens, as suggested in the OIE manual [17]. Furthermore, the mean death time (MDT) test was conducted on SPF chicks that were 9–10 days old, following the methods specified by Utami et al. [23].

## Results

### Isolation of NDV from rapid detection kit positive samples using ECEs

**Figure 2.**
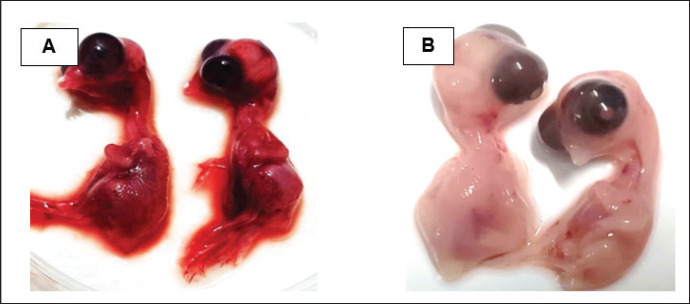
Characteristic changes in chicken embryo (9–10 day-old) after post-inoculation of NDV. (A) NDV virus-infected dead and hemorrhagic embryo; (B) Control embryo

They were using a rapid detection kit, and 87 out of 420 clinically suspicious samples (layers 38; Sonali 25; broilers 24) tested positive for the NDV antigen. Sixty of the 87 kit-positive samples were positive for NDV after being propagated in 9–10 days of seronegative ECEs (Table 1). The embryos inoculated with these samples exhibited mortality within 24 to 60 h post-inoculation, showing hemorrhagic lesions throughout their bodies. These hemorrhagic lesions were consistently observed in all parts of the deceased embryos when compared to control embryos (Fig. 2).

### Serological and molecular detection of isolated NDV

**Figure 3.**
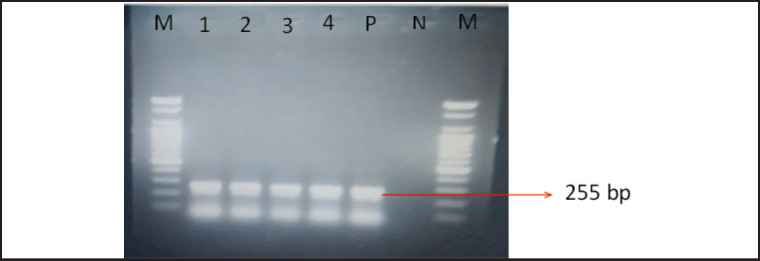
PCR amplification of F gene of NDV. M = 100 bp DNA marker, Lane 1–4 = field isolates of NDV, P = NDV positive control, N = negative control, M = 100 bp DNA marker.

Using NDV-specific hyperimmune serum, the HI test and AGID test clearly confirmed all 60 isolated samples as NDV. Each sample that yielded positive results in the HI and AGID tests underwent additional validation using RT-PCR. This test was confirmed by the presence of clear bands at the predicted size of 255 bp for the F gene (Fig. 3).

**Table 1.** Isolation of NDV from suspected field samples using ECEs

Outbreak Districts	No. of farms affected	No. of samples collected	Primary screening by rapid NDV Ag kit	Isolation of viruses using ECEs	HA test positive allantoic fluid	No of positive isolates of NDV using ECEs
Joypurhat	Sonali=7	42	12	12	8	10 (16.16%)
	Broiler=3	18	2	2	2	
Gaibandha	Layer=6	36	8	8	5	8 (13.13 %)
	Broiler=4	24	4	4	3	
Bogura	Sonali=5	30	8	8	5	8 (13.13 %)
	Broiler=5	30	4	4	3	
Sirajganj	Sonali=6	36	5	5	5	9 (15.0%)
	Broiler=4	24	5	5	4	
Tangail	Layer=7	42	16	16	8	10 (16.16%)
	Broiler=3	18	2	2	2	
Mymensingh	Layer=4	24	4	4	3	7 (11.66 %)
	Broiler=6	36	4	4	4	
Pabna	Layer=6	36	10	10	5	8 (13.13 %)
	Broiler=4	24	3	3	3	
**Total**	**70**	**420**	**87**	**87**	**60**	**60**

NDV-Newcastle diseases virus; ECEs-Embryonated chicken eggs; HA-Hemagglutination

### Analysis of phylogenetic tree

**Figure 4.**
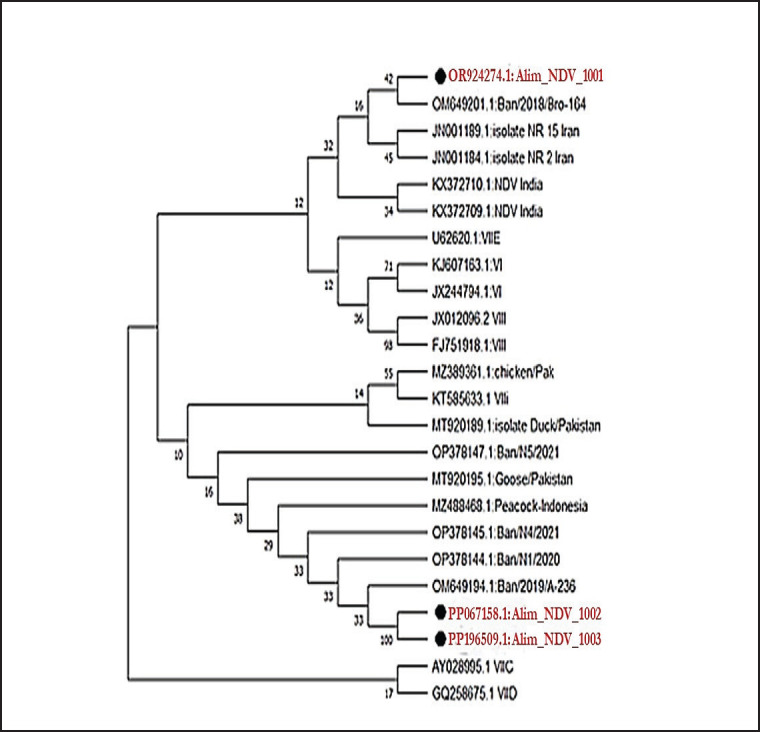
Phylogenetic analysis against the F gene of the recent isolates of NDV by the Neighbor-Joining method and the evolutionary investigations were carried out using the MEGA 11 program.

**Figure 5.**
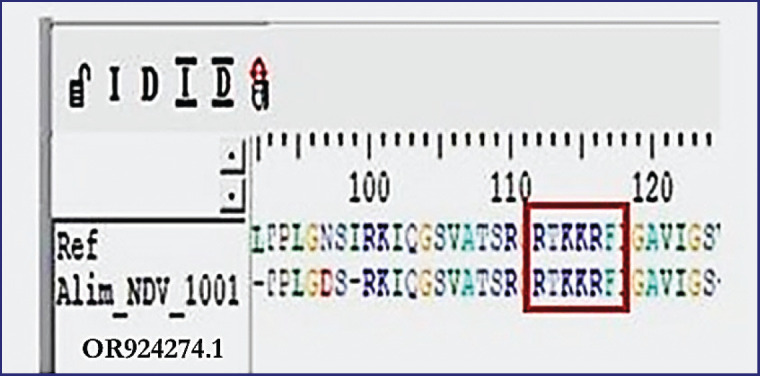
Amino acid sequence of the F protein cleavage site of the recent isolates denoted as ^112^-R-T-K-K-R-F-^117^, identifies them as velogenic NDV.

The phylogenetic investigation of the partial F gene nucleotide sequences shows the grouping of current strains of NDV under Genotype-VII. These sequences exhibit significant similarity with other strains identified in Bangladesh, Iran, and India, as depicted in Figure 4. Furthermore, the sequences of amino acids at the F protein cleavage site in the isolated strains, denoted as ^112^-R-T-K-K-R-^117^, identify them as velogenic NDV, as illustrated in Figure 5.

### HA pattern of isolated NDV with the blood of avian and mammalian species

The recent isolate of NDV exhibited a robust agglutination pattern when tested with the red blood cell (RBC) of chickens and domestic ducks. However, it displayed a weaker agglutination reaction with the RBCs of buffalo, cattle, guinea pigs, and humans. Interestingly, no agglutination was observed when testing geese, Muscovy ducks, sheep, goats, horses, and rabbits with the RBCs. To further analyze these HA patterns, we can compare them to the LaSota strain of NDV using RBCs from various avian and mammalian species, as shown in Table 2.

### Pathogenicity of isolated NDV

The latest isolate’s ICPI, IVPI, and MDT test results ranged from 1.56 to 1.75, 2.3 to 2.7, and 47.16 to 57.92 h, respectively. These results provide strong evidence that these isolates are velogenic strains of NDV.

## Discussion

Since poultry farming provides inexpensive animal protein, it is one of the most significant livestock-producing industries worldwide. However, ND is a prevalent and severe viral virus that significantly impacts the chicken industry [24]. The multiple lineages of NDV are circulating worldwide, and most of them are highly virulent. There have been a growing number of reports of outbreaks in vaccinated flocks worldwide, which can be attributed to the vaccine’s suboptimal protection. Even though live and inactivated vaccines against NDV were used, there was a severe outbreak of ND in 2020–21 in several vaccinated broiler, layer, and Sonali farms in different districts of Bangladesh.

**Table 2.** Hemagglutination patterns of NDV with RBC of different avian and mammalian species

RBC of avian and mammalian species	Observation			
	Recent isolates of NDV	Image of HA pattern	LaSota strain of NDV	Image of HA pattern
Chicken	**++++**	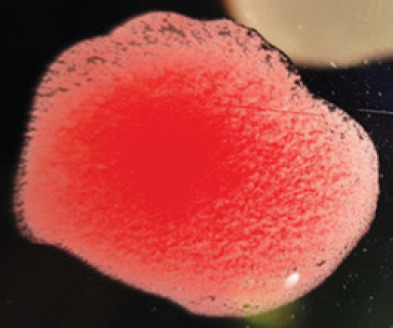	**++++**	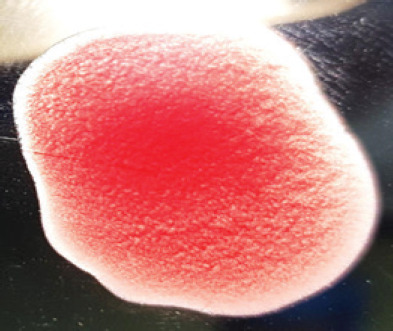
Domestic duck	**++++**	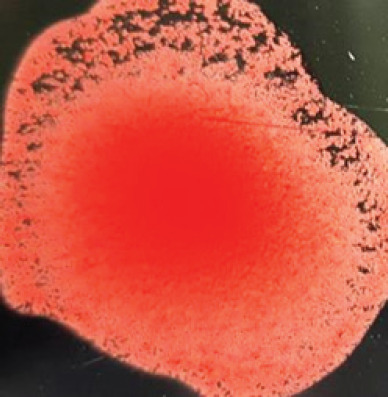	**++++**	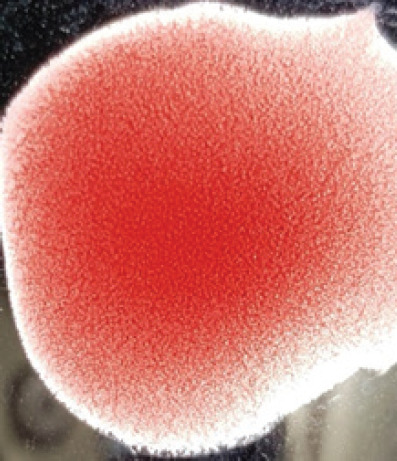
Goose	**--**	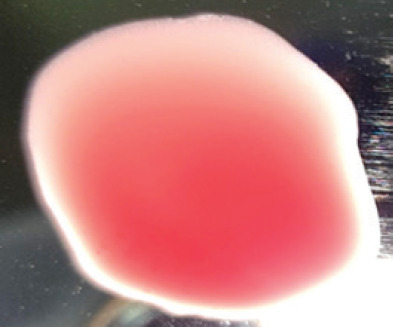	**++++**	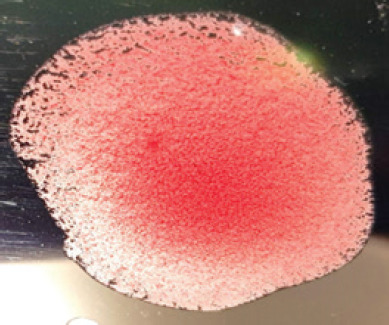
Muscovy	**--**	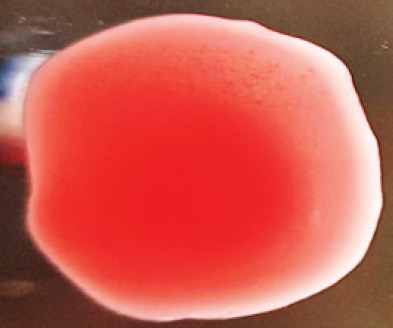	**++++**	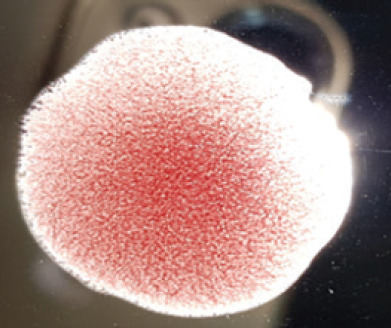
Cattle	**+++**	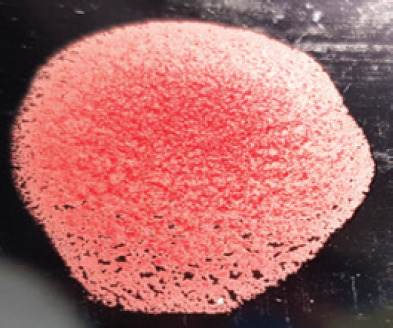	**--**	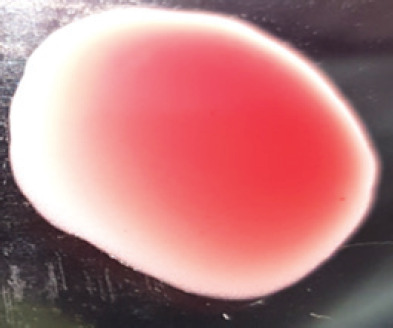
Buffalo	**+++**	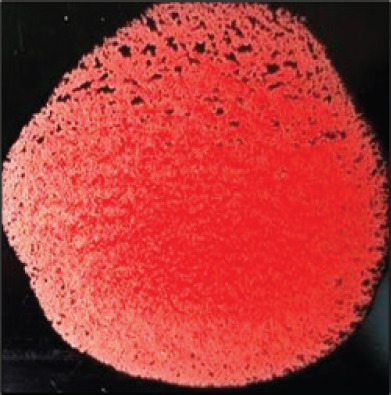	**+++**	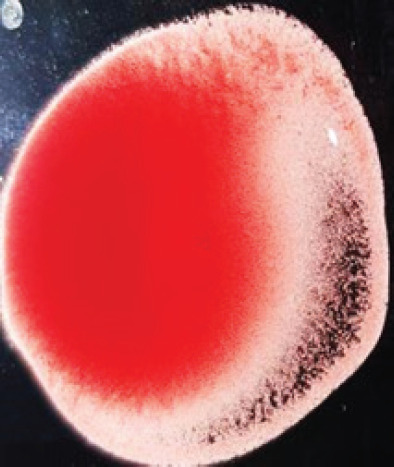
Sheep	**--**	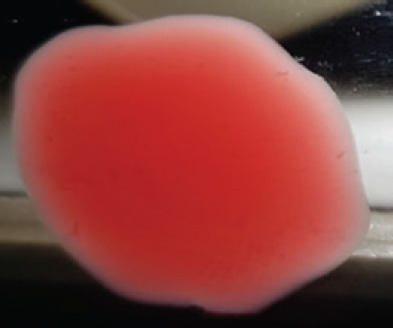	**+++**	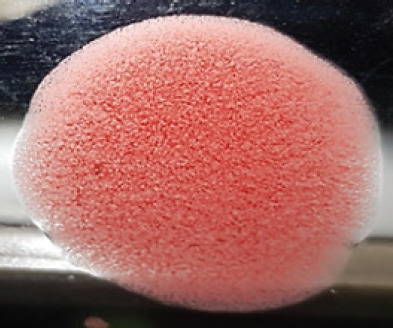
Goat	**--**	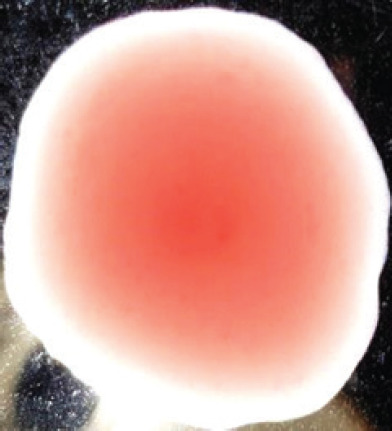	**--**	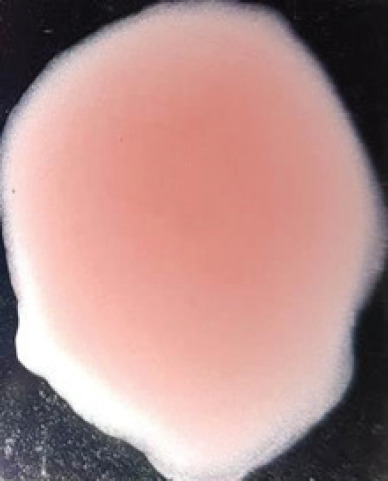
Horse	**--**	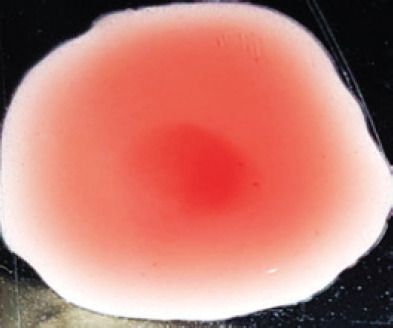	**--**	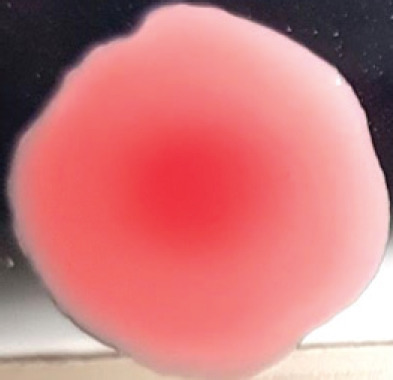
Rabbit	**--**	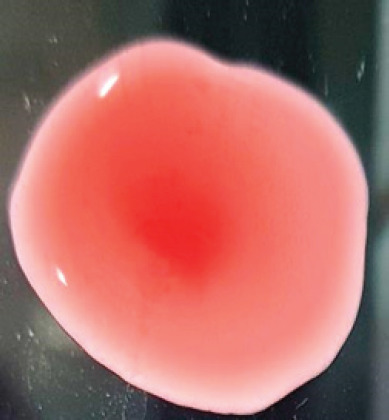	**--**	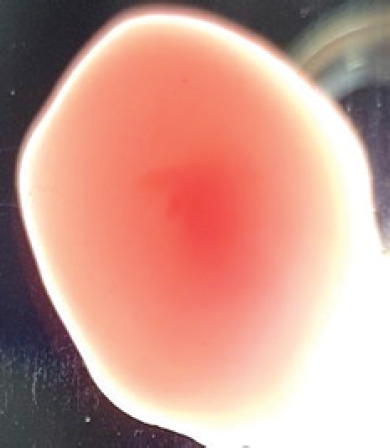
Guineapig	**+++**	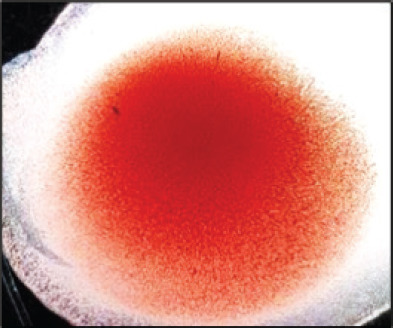	**+++**	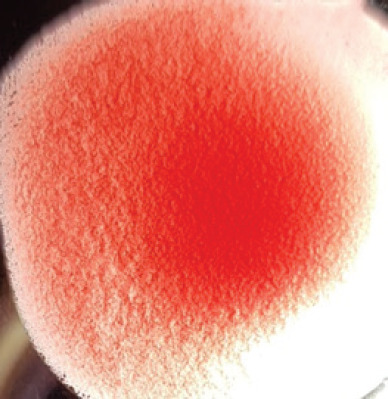
Human	**--**	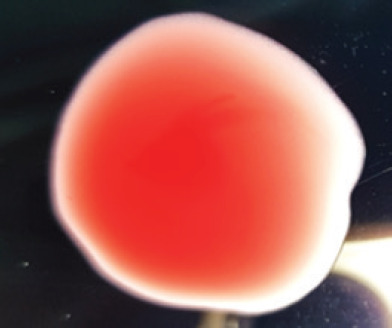	**++++**	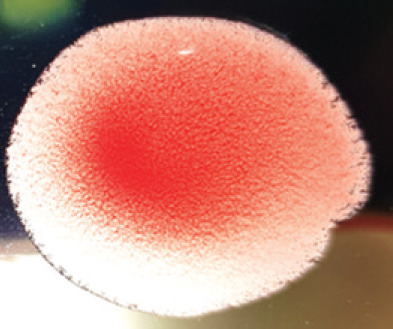

N.B. ++++ = Strong; +++ = Moderate; + = Weak; -- = Negative; RBC- Red blood cells; NDV-Newcastle diseases virus; HA-Hemagglutination

Sampling was done in recognized outbreak regions in Bangladesh. Most of the samples were collected from the chickens showing clinical signs of depression, coughing, greenish diarrhea, paralysis, twisted neck, soft-shelled eggs, stop of egg production, and death, which are closely comparable to the findings of Hasan et al. [25]. While sampling, the postmortem findings observed were small bleeding patches in the colon, ulcers with bleeding in the intestinal wall and cecal tonsils, congested and inflamed trachea, mucus discharge, and pin-point hemorrhages at the ends of proventricular glands [26]. The highest mortality was recorded in broiler birds (55%) of the 15–35-day age groups, and the lowest mortality was recorded in Sonali birds (25%) of the 1–30-day age groups. The mortality rate, clinical signs, and postmortem findings revealed that the isolates were very similar to virulent NDV [27].

Haque et al. [28] initially screened NDV from 150 cloacal swabs of chickens, pigeons, and quails using NDV rapid test kits. The screenings revealed that 96 (64%) out of the 150 samples tested positive for NDV, while 54 samples (36%) tested negative. Further analysis of the 96 positive samples showed that 77 (80.2%) were from chickens (with a distribution of 27 from broilers, 24 from layers, and 26 from native chickens), 12 (12.5%) from pigeons, and 7 (7.3%) from quails, indicating that the NDV prevalence was specifically observed in these bird species. The reason for the NDV rapid test kit’s failure to detect NDV in other field samples remains uncertain. However, it is speculated that additional respiratory diseases such as infectious laryngotracheitis, infectious bronchitis, or avian influenza virus could be responsible for the birds’ mortality. Similarly, the rapid detection method was employed for the preliminary screening of all 420 and was found positive in 87 samples. Bordoloi et al. [29], Duad et al. [30], and Ahmed and Odisho [31] utilized 9–10 days of seronegative chicken eggs for the propagation and isolation of the NDV virus from clinical field samples. Again, among the 87 screening-positive samples, 60 were successfully grown. In this study, seronegative 9–10-day ECEs were used for the isolation of NDV from the rapid kit, which was detected positive, and 60 out of 87 positive processed samples were found to grow in ECEs.

HI and AGID tests utilizing NDV-specific hyperimmune serum revealed that all 60 isolates tested positive for NDV. The HI test results of the current study support the findings of Ahmed and Odisho [31], El-Bagoury et al. [32], and Seal et al. [33], who confirmed NDV by the HI test. Hasan et al. [25] and Mohammed et al. [34] detected the NDV virus in field samples utilizing hyper-immune serum of NDV by AGID test.

The results of RT-PCR found that all 60 isolates had clear expected bands of 255 bp against the F gene of NDV. The results of the molecular detection of the F gene in the present study are highly similar to the findings of Li et al. [19], Tiwari et al. [35], and Wambura et al. [36]. F gene-based phylogenetic analysis also showed that the recent isolates of NDV belonged to genotype-VII and were closely associated with the other isolates of Bangladesh (OM649201), Iran (JN001189), and India (KX372710), having 99.7%–98.5% identity. The presence of the amino acid sequence ^112^-R-T-K-K-R-^117^ at the cleavage site of the F protein suggests that the most recent isolates belong to the velogenic strain of NDV. The findings of Elfatah et al. [37], Sultan et al. [38], and Munir et al. [39] showed almost similar types of F protein cleavage site motifs. The panzootic genotype VII.2 of NDV in chickens in Bangladesh is the cause of the first reported epidemic, according to Nooruzzaman et al. [7]. It also implies that this NDV genotype has recently returned to the area, maybe from Southeast or East Asia.

In the present study, the recent NDV isolates showed a strong agglutination pattern with chickens and domestic ducks; weaker reactions were observed with buffalo, cattle, guinea pigs, and humans, and no HA was observed with geese, Muscovy ducks, sheep, goats, horses, and rabbits. The results of the HA patterns of recent isolates of NDV also indicate that the circulating isolates of NDV are very virulent, which strongly agrees with the findings of Haruna et al. [40].

The ICPI, IVPI, and MDT assays confirmed that the isolates were velogenic strains of NDV, which is similar to the findings of Rashi et al. [41], Ahmed and Odisho [31], and Kommers et al. [42]. The MDT in chicken embryos and the ICPI in day-old chicks were two of the pathogenicity tests carried out by Nooruzzaman et al. [7]. The findings confirmed that the isolates from Bangladesh belong to the velogenic strain. When chickens that were 35 days old were given injections of two strains of NDV called LT67 and N5, all of them developed the disease within 3 days after the injections, and they all died by the 7th day. The autopsies showed significant bleeding, congestion, and tissue death in many organs, which is indicative of infections caused by the highly virulent viscerotropic strain of NDV.

## Conclusion

The study isolated the G-VII strain of Newcastle disease viruses from field samples from 2020–2021, confirming its highly virulent nature. The circulating strain may have been introduced to Bangladesh’s poultry population from Iran or India over the past decade. The frequent outbreaks in commercial poultry, including layer, Sonali, and broiler, are attributed to the new genotype of NDV. To successfully control ND in commercial poultry, it is necessary to develop a potent vaccine specifically designed for this new genotype.

## List of Abbreviations

AGID, Ager gel immune diffusion; ECEs, embryonated chicken eggs; HA, hemagglutination; HI, hemagglutination inhibition; ICPI, Intracerebral pathogenicity index; IVPI, Intravenous pathogenicity index; MDT, Mean death time; ND, Newcastle disease; NDV, Newcastle disease virus; OIE, Office international epizootics; RBC, Red blood cells; RT-PCR, Reverse transcription-polymerase chain reaction.
